# Lower back pain

**DOI:** 10.1093/emph/eou034

**Published:** 2015-01-10

**Authors:** Eric R. Castillo, Daniel E. Lieberman

**Affiliations:** Department of Human Evolutionary Biology, Harvard University, 11 Divinity Avenue, Cambridge, MA 02138, USA

## Lower back pain

Lower back pain (LBP) is one of the most common and costly medical problems today [1, 2]. Pain is usually transitory and can arise from the intervertebral discs, bones, ligaments and muscles of the spine. Risk factors for LBP include genetic, environmental, psychosocial and biomechanical influences [3]. However, although 85% of LBP cases have no clear etiology, 97% may be due to musculoskeletal issues [4]. Lumbar curvature (lordosis) is one factor that generates shearing between adjacent vertebrae and at intervertebral joints. People with high degrees of lumbar lordosis, including pregnant women, can experience excessive shearing (*F*_shear_) and compression (*F*_compression_) forces between lumbar vertebrae, most often between the last lumbar and the sacrum [3, 5]. In addition to other factors, including age-related spinal degeneration, high levels of *F*_shear_ and *F*_compression_ can lead to painful muscle strain, joint capsule pain, disc herniation, inflammation (spondylitis), bone degeneration (spondylolysis) and vertebral displacement (spondylolisthesis) [3–5].


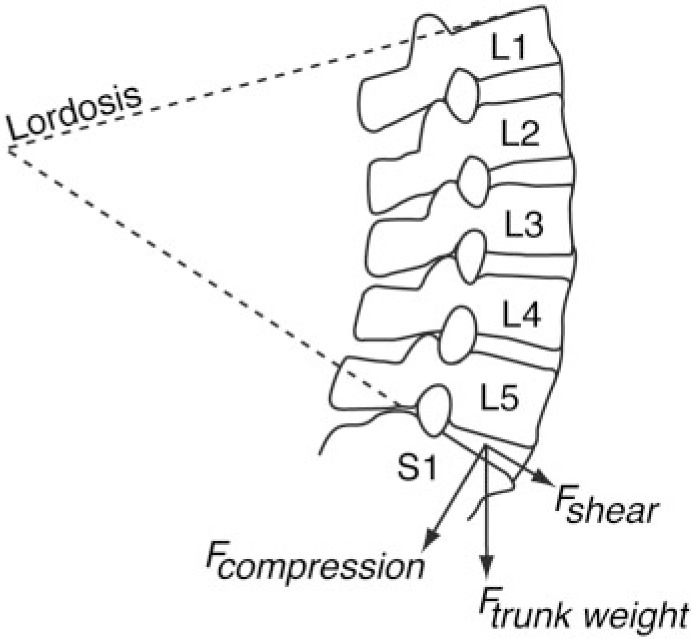


## Evolutionary perspectives

Mechanically induced LBP is often thought be a consequence of trade-offs in the spine due to selection for bipedalism from a quadrupedal ancestor. According to this hypothesis, the costs of increased *F*_shear_ due to lordosis were offset by the benefits of positioning the upper body’s center of mass over the hips, stabilizing the trunk and decreasing the costs of upright posture. The only two known complete lumbar spines from early hominins show the same sexually dimorphic pattern present in modern humans, with males having fewer wedge-shaped, lordotic vertebrae than females [5]. More fossils are needed, but this suggests selection for decreased *F*_shear_ in pregnant hominin females who exaggerate lordosis to cope with increased fetal mass. Another hypothesis is that some cases of LBP are the result of a recent mismatch, in which the modern human spine is poorly adapted to recent environmental conditions. Since hominins prior to the post-industrial era were very active [6], low levels of physical activity and abnormal spinal loading may result in weak, unstable back tissues and increased risk of pain and injury. Support for this comes from evidence that decreased back muscle strength and endurance strongly correlate with LBP [3]. In addition, novel behaviors that lessen loading, such as sleeping on soft mattresses and prolonged sitting in chairs, may be associated with higher LBP rates [7, 8]. Also, active farmers from low-income countries may have two to four times lower rates of LBP than sedentary, urban people from high-income countries, though demographic factors (e.g. age) may bias these findings [9].

## Future implications

If some cases of LBP are the result of a recent mismatch, LBP rates may be on the rise as sedentary behaviors increase. This may also suggest that trunk strengthening and endurance exercises can help treat and prevent some cases of LBP. However, the mismatch hypothesis has been poorly tested. More biomechanical research is needed to explore the relationship between novel types of spinal loading (e.g. sitting in chairs, using soft mattresses) and LBP. Detailed comparative studies of LBP rates around the world are necessary to test the hypothesis, comparing age-matched groups with different activity levels and subsistence patterns, such as hunter–gatherers and non-industrial populations.
